# Preparation of Polyamidoamine Dendrimer Modified Magnetic Nanoparticles and Its Application for Reliable Measurement of Sudan Red Contaminants in Natural Waters at Parts-Per-Billion Levels

**DOI:** 10.3389/fchem.2021.708995

**Published:** 2021-08-05

**Authors:** Yalin Wu, Huahua Bai, Qingxiang Zhou, Shuangying Li, Yayang Tong, Jinghan Guo, Boyao Zhou, Zhi Li, Yali Zhan, Menghua Liu, Yanhui Li, Tongxu Qu

**Affiliations:** ^1^ State Key Laboratory of Heavy Oil Processing, China University of Petroleum-Beijing, Beijing, China; ^2^ Beijing Municipal Research Institute of Environmental Protection, Beijing, China

**Keywords:** Sudan red dyes, magnetic solid phase extraction (MPSE), PAMAM dendrimer, PAMAM dendrimer-modified magnetic nanoparticles, high performance liquid phase chromatography

## Abstract

The health threat from Sudan red dyes has been the subject of much attention in recent years and is crucial to design and establish reliable measurement technologies. In the present study, a new magnetic nanomaterial, polyamidoamine dendrimer-modified magnetic nanoparticles (Gn-MNPs), was synthesized and characterized. The nanomaterials had good adsorption capacity for Sudan dyes from natural waters. G1.5-MNPs possessed excellent adsorption capacity and a linear adsorption relationship over the range from 0.02 to 300 μg L^−1^ of Sudan dyes with relative coefficients all larger than 0.996. The sensitivity of the proposed method was excellent with detection limits over the range from 1.8 to 5.5 ng L^−1^ and the precision was less than 3.0%. G1.5-MNPs showed a remarkable application potential for the enrichment of trace environment pollutants in aqueous samples and the developed method based on this nanomaterial could be a robust and reliable alternative tool for routine monitoring of such pollutants.

## Introduction

Sudan red I-IV dyes are well-known food additives that have been included in groupings of carcinogens (Yu et al., 2015; Liu et al., 2018). They are phenyl-azoic derivatives that are neurotoxic, genotoxic, and carcinogenic (Heydari et al., 2019; Moreno-González et al., 2020). Thus, Sudan dyes are forbidden in foodstuff at any concentration level for any purpose based on the regulations of the Food Standards Agency and European Union (Li et al., 2013a). Nevertheless, they are still attractive as food additives to enhance the appearance of chili, curry, ketchup, and Curcuma due to their bright red-orange color, colorfastness, and low cost (Sricharoen et al., 2017). There is, therefore, an urgent need to develop environmentally friendly, highly sensitive, effective, and practicable methods for monitoring Sudan Red pollutants.

Until recently various methods have been established based on enzyme-linked immunosorbent assay (ELISA), capillary liquid chromatography, high performance liquid chromatography with variable wavelength detector, liquid chromatography equipped with mass spectrometry, ultra-high performance liquid chromatography tandem mass spectrometry, (Chang et al., 2011; Chen et al., 2013; Zhou et al., 2014; Benmassaoud et al., 2017; Piątkowska et al., 2017). Among them, conversional liquid chromatography is widely used because of its low cost (Qi et al., 2011; Zhou et al., 2014). However, the residue concentration of pollutants is often very low and the sample matrix is complex, which makes it difficult to directly detect the target analytes. Therefore, sample pretreatment is of great value and has been a crucial step in the analytical process. At present, many sample pretreatment technologies have been explored for the enrichment of target pollutants including dispersive liquid-liquid microextraction (DLLME) (Zhou et al., 2014; Bazregar et al., 2018), liquid–solid extraction (Rebane et al., 2010), ultrasonic-assisted extraction (Yan et al., 2011), cloud point extraction (Liu et al., 2007), and solid-phase extraction (SPE) (Qi et al., 2011). Noticeably, SPE is regarded as a cost-effective tool due to its simplicity, high preconcentration, and low consumption of organic toxic solvents (Dibaei et al., 2016). Recently, there has been a growing interest in magnetic solid phase extraction (MSPE) due to its favorable properties including the small dosage of the adsorbent, simplicity, easy separation, and saving time (Gong et al., 2017; Li et al., 2014). As a reliable method, MSPE has been successfully used to preconcentrate environmental pollutants such as phthalate esters, polycyclic aromatic hydrocarbons, lignin, and heavy metal ions (Wu et al., 2017; Zhou et al., 2017; Wu et al., 2019; Yuan et al., 2020). MSPE is based on magnetic or magnetizable sorbents such as iron, cobalt, nickel, and their oxides. Among them, Fe_3_O_4_ is the most often-used core material due to its easy separation and reusability (Yan et al., 2017). It is crucial to functionalize it with different materials and to enhance its stability and extraction capacity (Li et al., 2013b).

Dendrimers, as the name implies, are a group of highly-branched polymers that contain three-dimensional architectures, and the molecular size, shape as well as function may be controlled precisely (Xiao et al., 2016). The structure of dendrimers is composed of three major parts which are initial core, interior branched repeating units, and exterior functional groups, respectively. As such, dendrimers own excellent properties such as viscosity, solubility, flexibility, and density distribution compared with polymers (Zarghami et al., 2016). Moreover, they can trap or encapsulate pollutants because of abundant empty cavities among the branches of dendrimers and high specific surface area (Sajid et al., 2018). Based on the literature, iron based magnetic core-support, silica-support, carbon-support, titania-support dendrimers have been used for the adsorption and removal of pollutants (Barakat et al., 2013; Wu et al., 2016). Amongst the dendrimers, PAMAM dendrimer is a significant class of dendrimers (Hayati et al., 2017). Our group has modified Fe_3_O_4_ with PAMAM dendrimer and developed sensitive determination methods for cadium and mercury, phthalate esters, and typical phenols from water, and the developed method provided exceptionable sensitivity and other merits such as simplicity, easy operation, and low cost (Wu et al., 2019; Wu et al., 2020; Yuan et al., 2020; Zhou et al., 2021).

The present work aimed to synthesize Fe_3_O_4_/polyamidoamine dendrimer (Gn-MNPs) composite via a simple grafting-to method, which possesses comprehensive advantages of toilless isolation of magnetic materials and high enrichment ability of dendrimers. The designative and synthetic materials were then used as MSPE adsorbents for the preconcentration of Sudan pollutants. The significant parameters, including generation of dendritic polymers, adsorbent amount, and other factors that would affect enrichment performance were investigated.

## Experimental

### Chemicals and Apparatus

(3-Aminopropyl) triethoxysilane (APTES, AR), methyl acrylate (MA, GC), Sudan Red dyes were bought from Aladdin Chemistry Co., Ltd. (Shanghai, China). ultrapure water was used in all experiments. The information of other reagents, analytical, and characterization instruments are discussed in detail in the supporting information of this article.

### Preparation of PAMAM Dendrimer-Modified Fe_3_O_4_ MNPs

**FIGURE 1:**
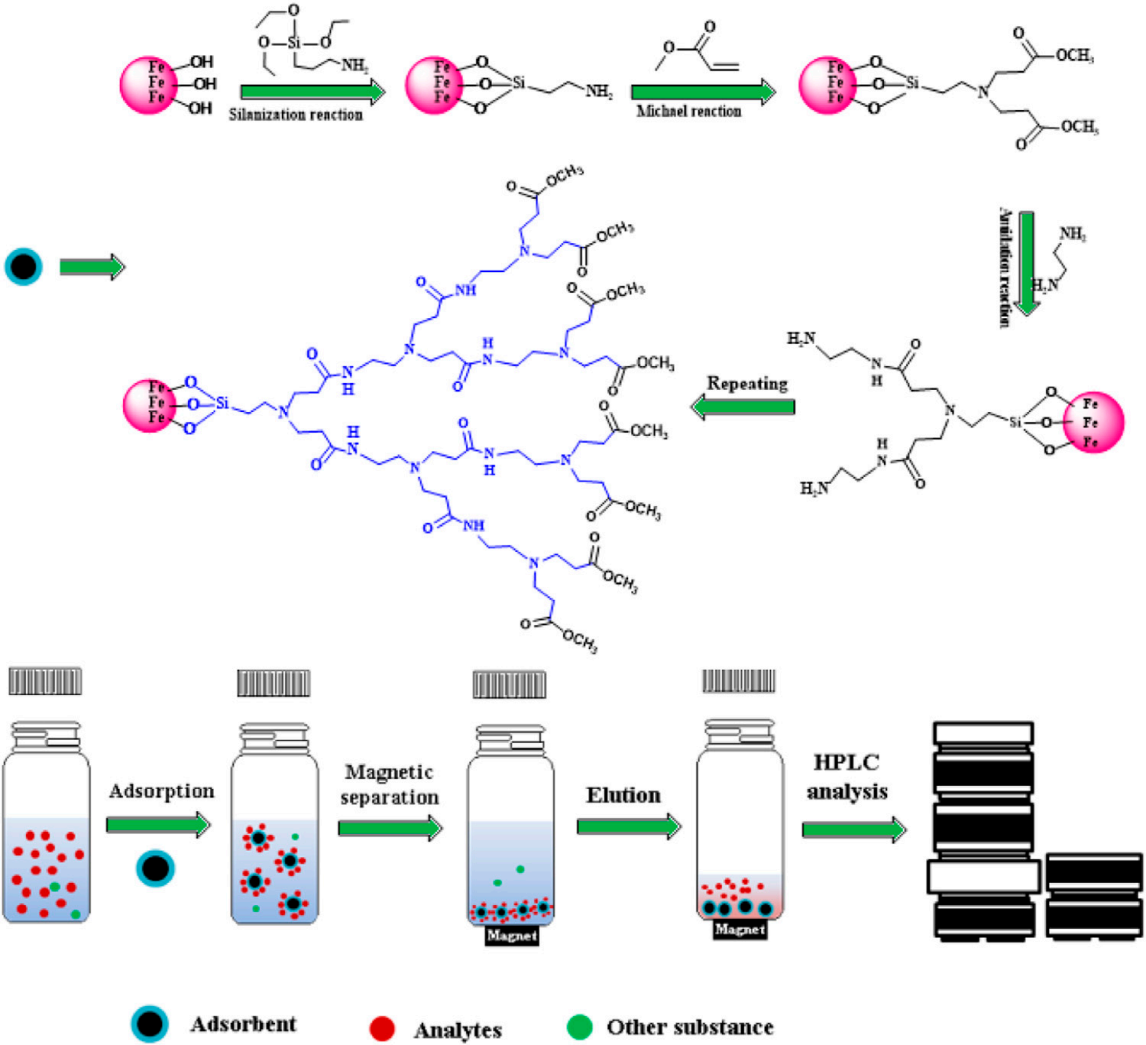
Schematic diagram of the preparation of Gn-MNPs.

Fe_3_O_4_ MNPs were prepared based on the work of Mardani (Mardani, 2017). Gn-MNPs were obtained based on a modified method (Kim et al., 2016). The synthesis of magnetic materials in detail is provided in supporting information. Figure 1 depicts the schematic diagram of the preparation of Gn-MNPs (n means generation number, generally, it is 0, 0.5, 1, 1.5, 2, 2.5) and adsorption of Sudan pollutants.

### Enrichment Process

First, 80 mg Gn-MNPs was diffused into a 50 ml spiked sample solution, and this solution was dispersed completely by stirring for 1 h. With putting a magnet, the phase separation was obtained and the adsorbent was collected. Then, Sudan red pollutants were desorbed from the adsorbent using 6 ml acetone in 9 min. Thereafter, the eluent was taken out and dried with a mild nitrogen stream. Finally, the residue was eluted and dispersed with 200 μl methanol. 50 μl of them was taken for analysis.

## Results and Discussion

### Characterization of MNPs and Gn-MNPs

**FIGURE 2:**
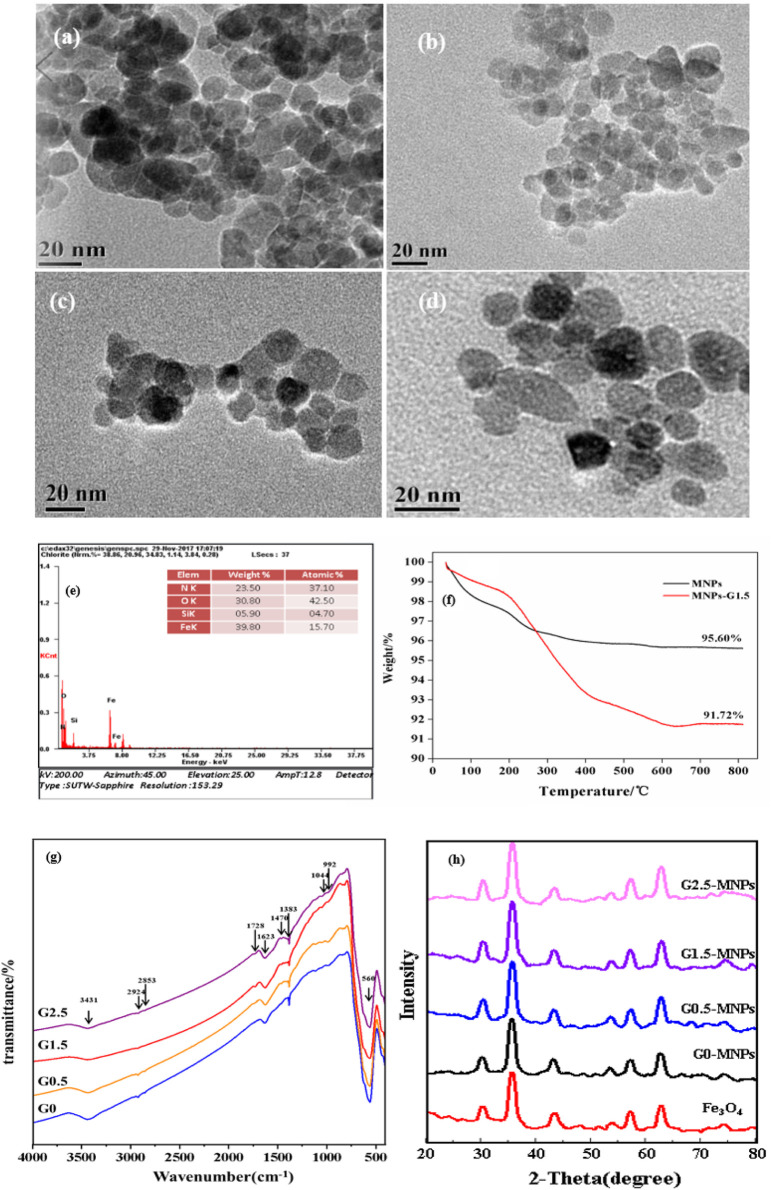
Characterization. **(A)** TEM image of Fe_3_O_4_ nanoparticles; **(B)** TEM image of G0.5-MNPs; **(C)** TEM image of G1.5-MNPs; **(B)** TEM image of G2.5-MNPs; **(E)** EDS image of G1.5-MNPs; **(F)** TGA of G1.5-MNPs; **(G)** FT-IR spectrums of Gn-MNPs; **(H)** XRD patterns of MNPs and Gn-MNPs.

Figure 2 depicts the morphology of naked MNPs and Gn-MNPs. Figures 2A,B,C,D demonstrates that MNPs were agglomerated which was ascribed to magnetic force between MNPs, while Gn-MNPs had better dispersion due to electrostatic stabilization as well as steric stabilization with the increase of PAMAM grafting generation. The average particle size of G1.5-MNPs was near 12 nm statistically. Moreover, the energy dispersive spectrum (EDS) of G1.5-MNPs is shown in Figure 2E. As revealed in Figure 2E, the materials contained iron, silica, oxygen, and nitrogen elements, which proved the success of synthesizing G1.5-MNPs.

Figure 2F shows the thermogravimetric weight change (TGA) curves of MNPs and G1.5-MNPs (Wu et al., 2020). The TGA curves diagrams indicate the weight loss of MNPs was about 4.4%, while G1.5-MNPs with a greater loss of approximately 8.3% was observed. The reason for the weight loss of naked MNPs was due to the physically adsorbed water and surface hydroxyl groups below 200°C. The weight loss of G1.5-MNPs increased owing to the decomposition of the dendrimers layer and the stable functional groups as the temperature changed from 200 to 800°C. In summary, we successfully synthesized adsorbent material.

Figure 2G illustrates the structure of as-prepared materials. The strong band at 560 cm^−1^ in all four samples was the typical characteristic of the vibration of the band between Fe and O, while the Gn-MNPs owed typical peaks at 1,044 and 992 cm^−1^ accounting for Si-O-Si and Si-O-Fe bonds, respectively, (Chou and Lien, 2010). Two feature peaks at 2,924 and 2,853 cm^−1^ were ascribed to the symmetry and asymmetry stretching-vibration of the -CH_2_- group, in addition, the absorption peak at 1,383 cm^−1^ was ascribed to the bending vibration of -CH_2_- group, which confirmed the existence of alkyl groups. Methyl group earns deformation vibration at 1,470 cm^−1^, which was not present in G0-MNPs. The broad peak at 3431cm^−1^ might mask the responses of the stretching vibration of -OH or -NH- group. The stretching vibration of and -CO-O- group and -CO-NH- group appear at 1728 and 1,623 cm^−1^, which is in agreement with the report from (Yuan et al., 2020). Furthermore, the response intensity raised at 1728 cm^−1^ due to the increase of the amount of -CO-O- groups as the dendrimer generation increased.

XRD patterns were shown in Figure 2H. Six evident characteristic peaks at about 2θ = 30.1, 35.1, 44.0, 53.9, 57.6,and 62.8 were due to crystal indexes of (220), (311), (400), (422), (511), and (440) of Fe_3_O_4_, which confirmed the cubic spinel phase of naked MNPs (Zhou et al., 2013). Moreover, Gn-MNPs and MNPs had analogical feature diffraction peaks, which proved that the progress of dendrimer modification did not alter the crystal phase of MNPs and MNPs were steady with magnetic property.

### Optimization of MSPE

**FIGURE 3:**
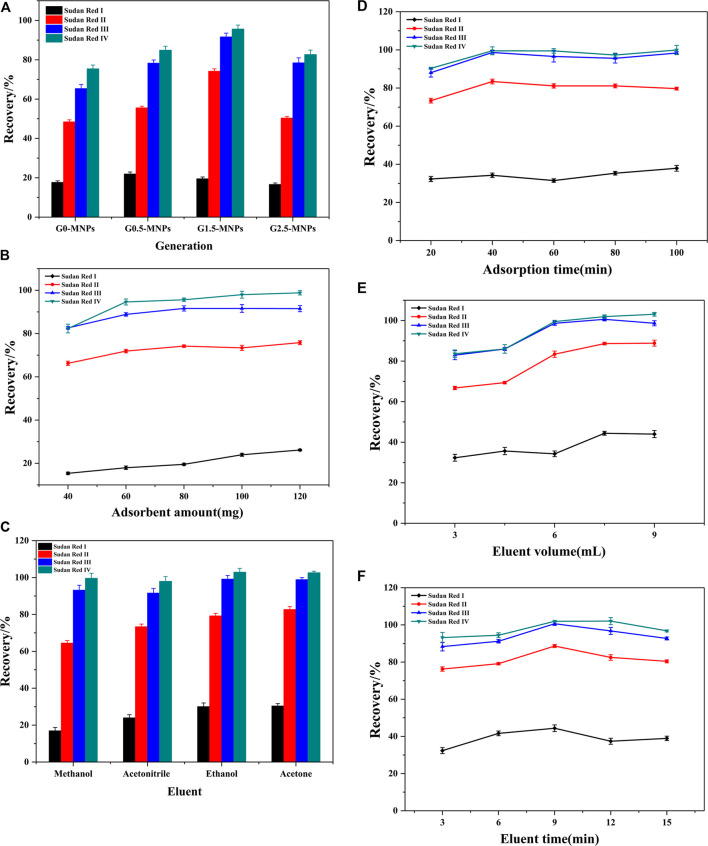
Optimization of enrichment parameters **(A)** Selection of PAMAM generation; **(B)** Effect of adsorbent dosage; **(C)** Effect of eluent; **(D)** Effect of adsorption time; **(E)** Effect of eluent volume; **(F)** Effect of elution time Experimental conditions: spiked level of analytes, 10 μg L^−1^; adsorbent amount, 80 mg; eluent, acetonitrile; adsorption time, 60 min; elution volume, 6 ml; elution time, 9 min; pH, seven; ionic strength, 0% (w/v); concentration of humic acid, 0 mg L^−1^; sample volume, 50 ml. Each parameter was optimized with other parameters keeping constant, and the optimal value was used in the subsequent optimization process.

Batch experiments were designed and performed to examine the extraction efficiencies of different generations of the magnetic material. Figure 3A depicts the extraction efficiencies of different generations of the prepared materials. It reveals that the extraction performance followed the order of G0-MNPs < G0.5-MNPs < G1.5-MNPs > G2.5-MNPs. There was a definite possibility that more active sites could be provided with the raise of PAMAM generation, while the generation number was higher than 1.5, the steric hindrance led to difficulty for target contaminants to enter the interior structure of PAMAM dendrimers, which led to the decline of the recoveries (Wu and Zhang, 2013). Consequently, G1.5-MNPs were the optical adsorbent for extraction.

As MSPE is concerned, the amount of adsorbent is an important parameter, which was tested from 40 to 120 mg. Figure 3B exhibits the effect of the amount of adsorbent. From the figure, it was obvious that the extraction performance of Sudan II-IV had a significant improvement along with the adsorbent dosage from 40 to 60 mg, and kept slow increase with further raising of the adsorbent amount from 60 to 100 mg and then kept constant with the further raise of the adsorbent dosage up to 120 mg. This was attributed to the fact that more adsorption sites were supplied with the increase in adsorbent dosage, and 100 mg adsorbents provided enough adsorption sites for the fixed spiked amount of analytes, which meant that the recoveries were almost unchanged when the amount was higher than 100 mg. However, the recoveries of Sudan I always increased slowly with further raising of the adsorbent amount in the range of 40–120 mg. Therefore, a constant amount of adsorbents (100 mg) was used in subsequent experiments.

Organic solvent with optimal elution performance will play a crucial role in the process of MSPE. To obtain an ideal elution performance, four different eluents such as methanol, ethanol, acetone, and acetonitrile were checked. Figure 3C describes the effect of the eluent. Among the four organic solvents, acetone resulted in the largest extraction efficiencies. The elution performance of ethanol was also very good, and a little lower than that with acetone. This may contribute to the fact that the more similar polarity between acetone and Sudan Red dyes, which made target analytes earn larger solubility in acetone according to the rule of like-dissolves-like. Hence, acetone was employed as the optimal eluent for the subsequent studies.

Adsorption is a complex procedure, and the adsorption rate to the adsorbent and desorption rate from the adsorbent are important in the extraction procedure. Good adsorption is obtained when a balance of the two rates is equal. Hence a reasonable time is crucial. The balance time was examined from 20 to 100 min. As can be seen from Figure 3D, the recoveries of Sudan pollutants raised with prolonging the time to 40 min and remained almost unchanged when the time changed from 40 to 100 min. It was certain that the extraction process had reached adsorption equilibrium when the adsorption time was at 40 min. Consequently, because of obtaining ideal extraction performance and shortening analytical time, the adsorption time was set at 40 min.

For the solid-phase extraction and MSPE procedures, eluting is an essential step. The volume of the elution solvent and desorption time play important roles in the elution process. These two factors were optimized by adjusting the elution volume from 3 to 9 ml and the elution time over the range of 3–15 min, respectively. As revealed by Figures 3E,F, 7.5 ml acetone could elute the Sudan red pollutants from nanomaterials and the elution procedure could be completed within 9 min, and there was no remarkable increase with further increase of the volume of elution solvent and elution time.

Sample pH is a crucial parameter that affects the mutual effects between the Sudan red pollutants and G1.5 MNPs adsorbent due to the different existing states of target analytes under different pH values. In this study, the sample pH was adjusted either by H_2_SO_4_ or NaOH solution. The results showed that good enrichment efficiencies were achieved under acidic and neutral conditions, while the recoveries decreased under alkaline conditions (the data were not provided). It was highly likely that the target Sudan red pollutants were apt to be decomposed and led to raising the solubility in water when the solution pH was higher than 7, which was not good for the enrichment of target Sudan red pollutants.

In general, the addition of salt into the samples improves the recoveries of the Sudan red dyes by affecting water solubility. A series of ionic strength experiments were carried out with the addition of NaCl from 5 to 20% (w/v) (Supplementary Figure 1). Supplementary Figure 1 reveals that there is a slight dip with the increase of NaCl concentration from 0 to 5% for Sudan II-IV, which could be attributed to the salting-in effect. Furthermore, the enrichment efficiencies rose with the raise of NaCl spiked from 5 to 20%, which was due to the effect of salting-out which reduced the solubility of target Sudan red pollutants in water with the increase of NaCl addition. It was noteworthy that the recoveries with a NaCl concentration at 20% (w/v) were higher than that with no addition of NaCl for Sudan II. The extraction performance of Sudan III and IV with 20% NaCl (w/v) was almost the same as that without the addition of NaCl. Unlike Sudan II-IV, the extraction performance of Sudan I had a constant and slow increase with the increase of NaCl in the range of 5–20%. Hence, NaCl was spiked at 20% (w/v) for all analytes in the following experiments.

Humic acid is a very common substance in natural waters, which sometimes has some impact on the enrichment of the pollutants with solid phase adsorbent. In the present study, it was investigated with concentrations varying from 0 to 20 μg ml^−1^ (Supplementary Figure 2). The recoveries of Sudan red pollutants sharply decreased with the addition of humic acid changing from 0 to 15% because of competitive adsorption, and then raised with the addition of humic acid, changing from 15 to 20 μg ml^−1^. The reason for this could be that Sudan red dyes were partially adsorbed onto the magnetic materials, which led to the increase of the recoveries. However, the extraction efficiencies with no humic acid were higher than with the addition of humic acid at 20 μg ml^−1^.

To estimate the influence of sample volume in the MSPE process, a series of experiments were performed in the range of 30–110 ml (Supplementary Figure 3). The extraction performance of Sudan III-IV increased with the increase of the sample volume in the range of 30–50 ml. Meanwhile, the extraction performance of Sudan I–II was constant. The extraction performance of Sudan red pollutants slightly decreased with sample volume larger than 50 ml. It was perhaps because the adsorbents were dispersed incompletely when the sample volume was too small, in addition, the amount of Sudan red pollutants increased with the sample volume rising from 50 to 110 ml, which meant that the fixed dosage of adsorbents could not provide enough active sites to completely adsorb the increasing analytes. Thus, the sample volume was optioned to be 50 ml.

### Evaluation of the Present Established Method

Under the aforementioned optimized conditions, the important analytical characteristics of the present established method are examined and the data are presented in Table 1. The data displayed that the linearity and LODs were respectively over the range of 0.02–300 μg L^−1^ (*R* ^2^, 0.996–0.999) and 1.8–5.5 ng L^−1^ (LOD, S/N = 3) for four Sudan dyes. The experimental results illustrate that the developed method is a feasible method for the measurement of Sudan dyes at low levels. Furthermore, the reproducibility was lower than 3.0% (*n* = 6), which indicated that the present established method owns satisfactory precisions.

**TABLE 1:** Analytical performances of methods.

Compound	Regression equation	Linear range (μg L^−1^)	*R* ^2^	Precisions (%, *n* = 6)	LOD (ng L^−1^)
Sudan I	y = 28.822x−90.641	0.02–300	0.998	1.4	4.9
Sudan II	y = 48.49x + 59.913	0.02–300	0.999	1.1	3.8
Sudan III	y = 63.975x + 213.09	0.02–300	0.996	3.0	1.8
Sudan IV	y = 64.606x + 201.28	0.02–300	0.996	2.8	5.5

### Real Water Sample Analysis

In this experiment, four water samples from the Dongsha river, Changping Park, Binhe Park, and Chaobai river were collected for estimating the possibility and practicality of this established method for real aqueous samples. These samples were pretreated and measured with the established method. The analytical results are presented in Table 2.

**TABLE 2:** Analytical results in real water samples.

Water sample	Spiked (μg L^−1^)	Recovery (%)			
		Sudan I	Sudan II	Sudan III	Sudan IV
Dongsha	0	nd^a^	nd	nd	nd
River	5	99.6^b^ ± 1.3^c^	96.4 ± 2.3	98.4 ± 2.2	98.9 ± 2.3
Water	10	100.7 ± 0.6	95.5 ± 0.8	95.8 ± 1.5	94.4 ± 1.4
Changping	0	nd	nd	nd	nd
Park	5	96.2 ± 1.6	95.1 ± 1.2	97.0 ± 0.6	99.3 ± 0.7
Water	10	98.7 ± 2.3	102.2 ± 4.0	99.2 ± 1.4	99.2 ± 2.1
Binhe	0	nd	nd	nd	nd
Park	5	97.9 ± 4.6	95.5 ± 1.5	99.4 ± 1.1	98.8 ± 1.3
Water	10	99.8 ± 0.9	98.9 ± 1.2	98.0 ± 2.0	97.7 ± 2.0
Chaobai	0	nd	nd	nd	nd
River	5	101.7 ± 3.7	96.7 ± 4.4	92.5 ± 3.0	93.7 ± 3.2
Water	10	98.3 ± 1.6	98.5 ± 1.7	97.8 ± 2.8	98.1 ± 2.6

a Not detected.

b Mean of three determinations.

c Standard deviation for three determinations.

In the blank samples, Sudan dyes were not detected in the collected aqueous samples. For validating the present established method, the real water samples were fortified with Sudan red pollutants at two different concentrations of 5 and 10 μg L^−1^. The mean recoveries were satisfied over the range from 92.5 to 102.2%. These data demonstrated that the proposed method provided satisfactory recoveries and precision for the reliable measurement of these pollutants in aqueous samples.

A comparison was made between the analytical performances of the established method with the existing methods reported (Table 3). The present established method had a much wider linear scope and lower LODs than most of the previously reported examples. Besides, lower RSDs in the present work demonstrated that satisfactory repeatability was achieved. On the whole, the MSPE with G1.5-MNPs as adsorbent prior to HPLC-UV shows superiority over other methods and it is an ideal extraction and analytical method for trace Sudan dyes in natural waters.

**TABLE 3:** Comparison with reported methods for the determination of Sudan dyes.

Analytical Method	Matrice	Linear range (μg L^−1^)				LOD (μg L^−1^)	RSD (%)	Ref
		Sudan I	Sudan II	Sudan III	Sudan IV			
MALLME^a^-LC-DAD	Juice	4.50−300	4.50−300	4.50−250	4.50–250	1.08–1.30	4.8–6.0	Hu et al. (2016)
MA-HILME^b^-HPLC-UV	Red wines	0.5−100	0.5−100	0.5−100	0.5–100	0.16–0.24	4.2–4.8	Song et al. (2015)
DLLME^c^-HPLC-UV	Water	0.3−40	0.3−40	1.2−160	1.2–160	0.18–0.46	3.7–5.9	Zhou et al. (2014)
MSPE-UFLC^d^-UV	Water	0.40−40	0.40−40	0.40−40	0.40–30	0.082–0.12	1.9–5.0	Wang et al. (2013)
MSPE-HPLC-UV	Water	0.025−5	0.025−5	0.025−5	0.025–5	0.0029–0.0073	2.5–6.9	(Li et al., 2013)
MSPE-UFLC-UV	Water	0.10−50	0.10−50	0.50−50	0.50–50	0.066–0.12	2.7–8.6	Jiang et al. (2012)
MSPE-HPLC-UV	Water	0.02−300	0.02−300	0.02−300	0.02–300	0.0018–0.0055	1.1–3.0	Present work

a Microwave-Assisted Liquid-Liquid Microextraction.

b Microwave-Assisted Homogeneous ionic Liquid Microextraction.

c Dispersive liquid-liquid Microextraction.

d Ultra Fast Liquid Chromatography.

## Conclusion

In the present study, polyamidoamine (PAMAM) dendrimer was successfully modified onto Fe_3_O_4_ nanoparticles, and a novel magnetic solid phase extraction with 1.5 generation of PAMAM dendrimer modified magnetic nanomaterials for the enrichment of trace Sudan Red contaminants was developed. The effective method based on MSPE as an adsorbent in combination with HPLC-UV for the sensitive measurement of Sudan red pollutants in real waters was successfully established. The proposed method showed excellent linearity ranges, low LODs, and good precision. Moreover, the established MSPE-HPLC-UV method demonstrated that it was very simple, fast, easy to separate, and cost was very low, which can match with the demand of pre-concentration and measurement of trace Sudan red pollutants and could be a good alternative for the analysis of pollutants in an aqueous sample.

## Data Availability

The raw data supporting the conclusions of this article will be made available by the authors, without undue reservation.
