# Supplementary material for: Preparation of Polyamidoamine Dendrimer Modified Magnetic Nanoparticles and Its Application for Reliable Measurement of Sudan Red Contaminants in Natural Waters at Parts-Per-Billion Levels
Source: Front Chem. 2021 Aug 5;9:708995. doi: 10.3389/fchem.2021.708995 (PMC8374313; doi:10.3389/fchem.2021.708995)
Supplement: Supplementary file 1 [file Presentation1.pdf]

Preparation of polyamidoamine dendrimer modified magnetic nanoparticles and its application for reliable measurement of Sudan red contaminants in natural waters at parts-per-billion levels

Yalin Wu[a,b], Huahua Bai[b], Qingxiang Zhou[a]*, Shuangying Li[a], Yayang Tong[a], Jinghan Guo[a], Boyao Zhou[a], Zhi Li[a], Yali Zhan [a]*, Menghua Liu[a], Yanhui Li[a], Tongxu Qu[a]

[a]*State Key Laboratory of Heavy Oil Processing, College of Chemical Engineering and Environment, China University of Petroleum (Beijing), Beijing 102249, China*

[b]*Beijing Municipal Research Institute of Environmental Protection, Beijing 100037, China*

1.1 Chemicals and apparatus

Ferric chloride hexahydrate ($FeCl_3·6H_2O$, AR) was purchased from Sinopharm Chemical Reagent Co., Ltd (Shanghai, China). Ferrous sulfate heptahydrate ($FeSO_4·7H_2O$, AR) was obtained from Modern Oriental Technology Development Co., Ltd (Beijing, China). (3-Aminopropyl) triethoxysilane (APTES, AR), methyl acrylate (MA, GC), Sudan Red dyes (I, II, III, IV) were all obtained from Aladdin Chemistry Co., Ltd (Shanghai, China). Ammonium solution ($NH_3·H_2O$, AR) and ethylenediamine (EDA, AR) were supplied from Tianjin Fuchen Chemical Reagent Factory (Tianjin, China). Humic acid (HA, CP), sodium hydroxide (NaOH, AR), and sodium chloride (NaCl, AR) were purchased from Tianjin Guangfu Fine Chemical Research Institute

(Tianjin, China). Methanol (AR), ethanol (AR), acetone (AR), and sulfuric acid (H_2SO_4, AR) were obtained from Beijing Chemical Works (Beijing, China). Methanol and acetonitrile (ACS/HPLC grade) were obtained from J&K Scientific Co., Ltd (Beijing, China). All reagents were used as received without further purification and ultrapure water was throughout all experiments.

An Agilent 1260 HPLC system with an ultraviolet variable wavelength detector (VWD) (Santa Clara, CA, USA) was used for analysis. An InertSustain C18 column (250 × 4.6 mm, 5 μm) was used for separation, and the mobile phase was composed of acetonitrile and water (95:5, v/v). The flow rate was controlled at 1.0 mL min^{-1}. The detection wavelength was set at 478 nm before six minutes and at 520 nm after six minutes, respectively. Transmission electron microscopy (TEM) images were obtained from a JEM-2100 Transmission Electron Microscope (Japan). Fourier-transform infrared spectroscopy (FT-IR) measurement was carried out on a MAGNA-IR 560 E.S.P spectrometer (Madison, Wisconsin, USA). X-ray diffraction (XRD) was measured with a Bruker D8 Advance Diffractometer (Germany).

1.2 Preparation of PAMAM dendrimer-modified Fe_3O_4 MNPs

Fe_3O_4 MNPs were prepared as follows:

2.78 g $FeSO_4·7H_2O$ and 5.40 g $FeCl_3·6H_2O$ were dissolved in 100 mL deoxygenated water. $NH_3·H_2O$ was dropwise added into the above three flasks under vigorous mechanical stirring until the pH was about 10. Then, the mixture was heated at 80 °C for 30 min. After that, the particulate materials were collected by a magnet and washed with water several times. Finally, the obtained materials were dried in a vacuum at 50 °C overnight.

Gn-MNPs were synthesized as follows:

2 g Fe_3O_4 was dispersed in 200 mL ethanol, and further the mixture was ultrasonicated and stirred for 30 min. Afterward, 14 mL APTES was dropwise added into the mixture under vigorous mechanical agitation. The mixture was then heated at 60 °C for 7 h. Eventually, the black powder was collected by a magnet and washed with ultrapure water and ethanol several times and dried in a vacuum at 50 °C overnight. The obtained materials were regarded as G0-MNPs.

40 mL of G0-MNPs methanol solution was ultrasonicated and stirred for 30 min to produce a finely dispersed suspension. Then, 72 mL methyl acrylate (MA) methanol solution (44%, v/v) was dropwise dropped to the above mixture. The mixture was then ultrasonicated for 7 h at room temperature. The product was separated with a magnet and washed with methanol five times. Finally, the obtained materials were dried in a vacuum at 50 °C overnight and regarded as G0.5-MNPs.

15 mL of G0.5-MNPs methanol solution was ultrasonicated and stirred for 30 min. Then, 45 mL EDA methanol solution (66%, v/v) was dropwise added to the mixture. After ultrasonically reacting at room temperature for 3h, the product was collected by a magnet and washed with methanol five times. Finally, the achieved materials were dried in a vacuum at 50 °C overnight and regarded as G1.0-MNPs. The procedure was repeated to gain the desired Gn-MNP (n= 1.5, 2.5), Nitrogen gas flow was used throughout the whole process.

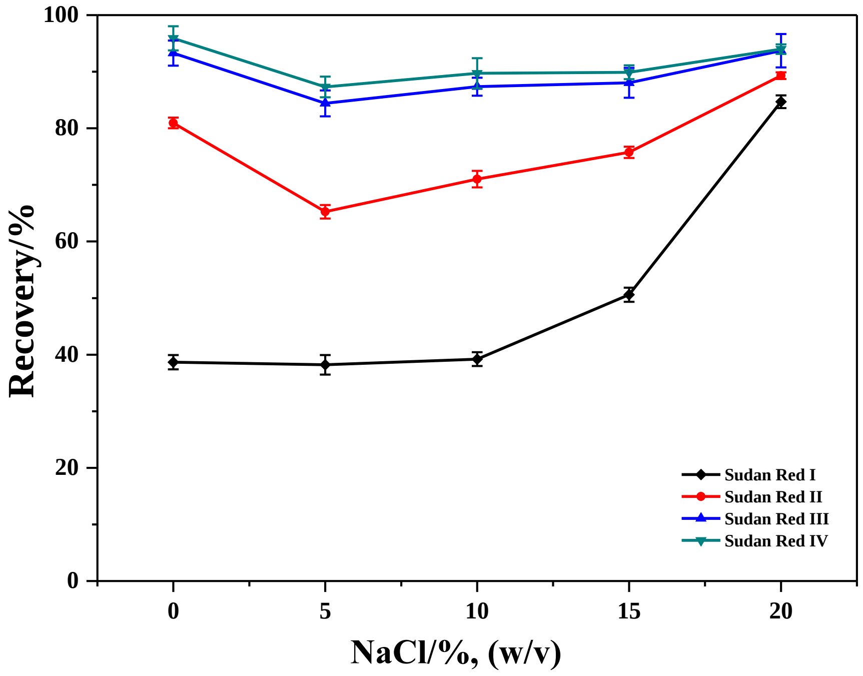

Figure S1. Optimization of ionic strength

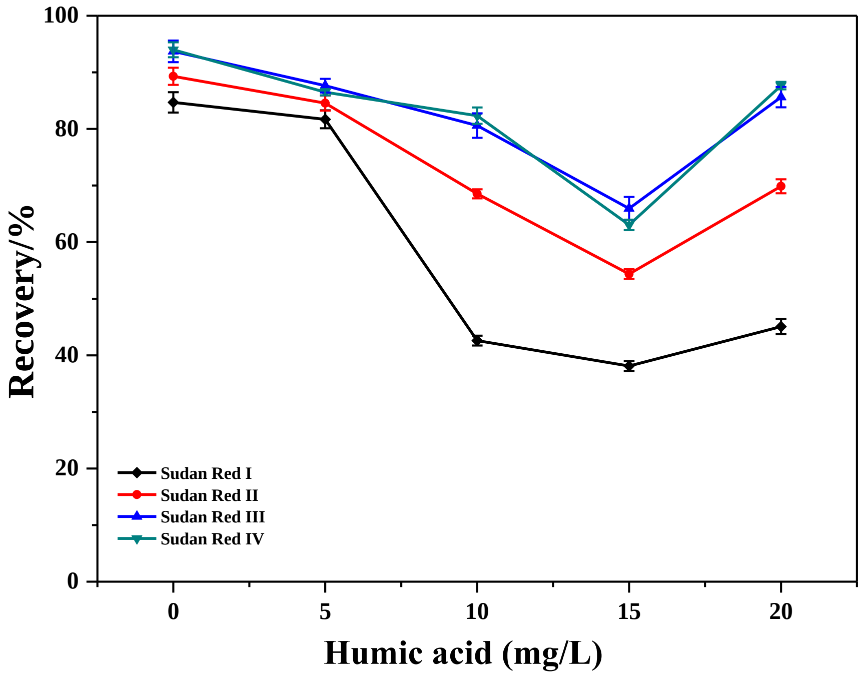

Figure S2. Optimization of humic acid

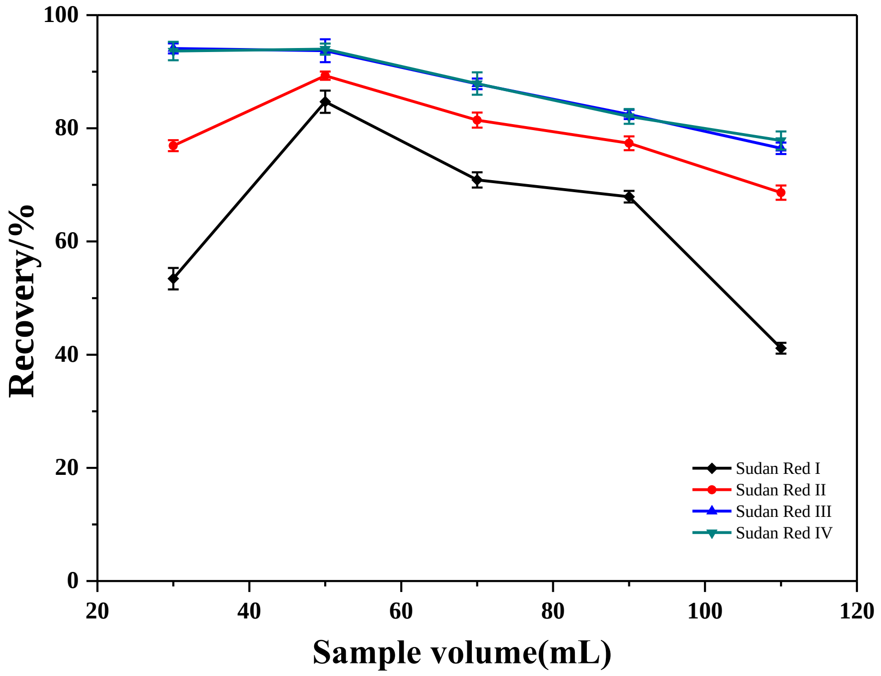

Figure S3 Optimization of sample volume
